# Lauric Acid Production in a Glycogen-Less Strain of *Synechococcus* sp. PCC 7002

**DOI:** 10.3389/fbioe.2015.00048

**Published:** 2015-04-24

**Authors:** Victoria H. Work, Matthew R. Melnicki, Eric A. Hill, Fiona K. Davies, Leo A. Kucek, Alexander S. Beliaev, Matthew C. Posewitz

**Affiliations:** ^1^Civil and Environmental Engineering Division, Colorado School of Mines, Golden, CO, USA; ^2^Microbiology Group, Pacific Northwest National Laboratory, Richland, WA, USA; ^3^Department of Chemistry and Geochemistry, Colorado School of Mines, Golden, CO, USA

**Keywords:** cyanobacteria, turbidostat photobioreactor, fatty acid secretion, metabolic overflow, organic acids, *glgC*, nitrogen deprivation, dodecanoic acid

## Abstract

The cyanobacterium *Synechococcus* sp. Pasteur culture collection 7002 was genetically engineered to synthesize biofuel-compatible medium-chain fatty acids (FAs) during photoautotrophic growth. Expression of a heterologous lauroyl-acyl carrier protein (C12:0-ACP) thioesterase with concurrent deletion of the endogenous putative acyl-ACP synthetase led to secretion of transesterifiable C12:0 FA in CO_2_-supplemented batch cultures. When grown at steady state over a range of light intensities in a light-emitting diode turbidostat photobioreactor, the C12-secreting mutant exhibited a modest reduction in growth rate and increased O_2_ evolution relative to the wild-type (WT). Inhibition of (i) glycogen synthesis by deletion of the *glgC*-encoded ADP-glucose pyrophosphorylase (AGPase) and (ii) protein synthesis by nitrogen deprivation were investigated as potential mechanisms for metabolite redistribution to increase FA synthesis. Deletion of AGPase led to a 10-fold decrease in reducing carbohydrates and secretion of organic acids during nitrogen deprivation consistent with an energy spilling phenotype. When the carbohydrate-deficient background (Δ*glgC*) was modified for C12 secretion, no increase in C12 was achieved during nutrient replete growth, and no C12 was recovered from any strain upon nitrogen deprivation under the conditions used. At steady state, the growth rate of the Δ*glgC* strain saturated at a lower light intensity than the WT, but O_2_ evolution was not compromised and became increasingly decoupled from growth rate with rising irradiance. Photophysiological properties of the Δ*glgC* strain suggest energy dissipation from photosystem II and reconfiguration of electron flow at the level of the plastoquinone pool.

## Introduction

Photosynthetic metabolism generates a wide range of biomolecules fundamental to energy, agriculture, and health (Durrett et al., 2008; Hu et al., 2008; Atsumi et al., 2009; Lubner et al., 2009; Lindberg et al., 2010; Lu, 2010; Niederholtmeyer et al., 2010; Kilian et al., 2011; Wahlen et al., 2011; Ducat et al., 2012; Elliott et al., 2012; Work et al., 2013; Sorek et al., 2014). Having rapid growth rates, efficient energy conversion, and metabolic adaptability, photosynthetic microorganisms (PSMs) including genetically tractable unicellular algae and cyanobacteria have received substantial attention for synthesizing biofuel precursors via native or transgenic processes (Ferrari et al., 1971; Cascon and Gilbert, 2000; Heifetz, 2000; Lee, 2001; Schmer et al., 2008; Rodolfi et al., 2009; Elliott et al., 2011; Fore et al., 2011; Liu et al., 2011; Radakovits et al., 2011; Soratana and Landis, 2011; Rosgaard et al., 2012; Bentley et al., 2013; Gronenberg et al., 2013; Leite et al., 2013; Möllers et al., 2014; Davies et al., 2015).

Biodiesel can be derived from biological fatty acids (FAs) extracted from photosynthetic organisms (Ma and Hanna, 1999; Durrett et al., 2008; Hu et al., 2008). While oil content and quality differs between species, the composition of FAs typically includes the predominant 16- and 18-carbon FAs (C16 and C18), as well as varying levels of shorter (C8–C14) and longer (≥C20) FAs (Gopinath et al., 2010). Currently, vegetable oil is the main source of the approximately 20 billion liters (L) of biodiesel produced yearly worldwide (Hoekman et al., 2012; Kopetz, 2013). However, recent efforts in genetic engineering seek to utilize microorganisms for FA production, a concept that would transition fuel production from cropland to bioreactors or pond systems (Lee, 2001; Hu et al., 2008; Li et al., 2008; Rodolfi et al., 2009; Leite et al., 2013). Lauric acid (C12:0) is naturally synthesized by coconut, palm, and bay trees (Litchfield et al., 1967; Denke and Grundy, 1992) and, when esterified, exhibits qualities comparable to modern diesel fuel, with better cold-flow properties relative to longer chain FAs (Gopinath et al., 2010; Hoekman et al., 2012). Microbial C12 synthesis has been achieved via transgenics in both heterotrophic and photoautotrophic hosts (Ohlrogge et al., 1995; Lu et al., 2008; Liu et al., 2011; Radakovits et al., 2011; Lennen and Pfleger, 2012), offering diverse opportunities in production platforms.

In photosynthetic eukaryotes, FAs of specific chain lengths are hydrolyzed from acyl carrier protein (ACP) by thioesterase enzymes, and the released FAs move out of the chloroplast into the cytoplasm where they are activated by coenzyme A (CoA) to facilitate transfer into higher lipids (Radakovits et al., 2010; Li et al., 2013). It has been found that many bacteria, including cyanobacteria, typically bypass the free FA (FFA) intermediate when assembling newly synthesized FA into membrane lipids (Sato and Wada, 2010; Jansson, 2012). When heterologous thioesterases are expressed in certain bacteria, most of the hydrolyzed FFA is found either in the culture medium or associated with the outside of the cell (Voelker and Davies, 1994; Ohlrogge et al., 1995; Liu et al., 2011; Zhang et al., 2011; Ruffing and Jones, 2012; Ruffing, 2014). Though the mechanism of secretion is not established, it is known that extracellular FFA can move back across the membrane and be reincorporated into metabolism by an acyl–acyl carrier protein synthetase (AAS) (Kaczmarzyk and Fulda, 2010). If this enzyme is disrupted, FFAs remain in the medium and can separate from the aqueous cultures. In the present study, carbon distribution in the cyanobacterium *Synechococcus* sp. Pasteur culture collection (PCC) 7002 was modified for C12 FFA synthesis by heterologous thioesterase expression (and AAS deletion) in both wild-type (WT) and carbohydrate-deficient genetic backgrounds.

## Materials and Methods

### Genetic engineering of *Synechococcus* sp. PCC 7002

*Synechococcus* sp. PCC 7002 (*Synechococcus* sp. 7002 hereafter) was genetically modified using previously described protocols (Frigaard et al., 2004; Xu et al., 2011). The pAQ1Ex plasmid containing the *Synechocystis* sp. 6803 promoter *cpcBA* (Xu et al., 2011) and spectinomycin antibiotic-resistance gene *aadA* (Frigaard et al., 2004); and the kanamycin-resistant Δ*glgC* mutant (Guerra et al., 2013) harboring the gene *aphII* (Frigaard et al., 2004) were kindly provided from the laboratory of Donald A. Bryant. In *Synechococcus* sp. 7002, the gene *glgC* (NC_010475.1) encodes ADP-glucose pyrophosphorylase (AGPase), which activates glucose for polymerization. The gene *fadD* (NC_010475.1) encodes a putative AAS with homology to the *Synechocystis* sp. PCC 6803 gene *slr1609* (NC_000911.1) (Kaczmarzyk and Fulda, 2010; Gao et al., 2012). A thioesterase derived from *Umbellularia californica* encoded by the gene *fatB1* (GenBank M94159) hydrolyzes 12-carbon FA chains from ACP during FA synthesis yielding lauric acid (C12), and a version of this gene codon optimized for expression in *Synechocystis* sp. 6803 was generously provided from the laboratory of Roy Curtiss III (Liu et al., 2011). Lauric acid and C12 in the text designate transesterifiable 12-carbon saturated fatty acyl chains.

The pAQ1Ex vector was modified for knockin expression of *fatB1* concurrent with deletion of the putative AAS. To construct the lauric acid secretion (LAS) module, *fatB1* was placed between the vector’s promoter and antibiotic selection marker via *Nco*I/*Bam*HI restriction sites. Flanking sequences of the *fadD* gene were inserted to target the cassette for homologous recombination using F||R primer pairs (5′–3′) 1:gttcacATGCATggctaggttcgtaatctttgggggta||gtatagGAATTCgccgaaatcatggctacaatcctacttt, 2:catactGTCGACgatccgaatggcggaatcttcg||gttcacGCATGCgtgctggcttttgtcacaatcttcttg (restriction enzyme recognition sequences used in plasmid construction are capitalized). Transformation was accomplished following an established protocol for homologous recombination in this organism (Xu et al., 2011). Integration of the LAS module into all genome copies was achieved by increasing spectinomycin pressure and confirmed by PCR (not shown) for complete allele segregation using the 1F and 2R primers listed above. The strain SA01 contains the LAS module in a WT background, and the strain SA13 contains this module in a carbohydrate-deficient background (Table 1).

**Table 1:** ***Synechococcus* sp. 7002 strains used in this study**.

Strain	Description	Genotype
WT	Wild-type *Synechococcus* sp. 7002	
SA01	Secretes lauric acid^a^	*ΔfadD::*P*_cpcBA_-fatB1-aadA*^b^
Δ*glgC*	RC-deficient (AGPase disrupted)	*ΔglgC::aphII*
SA13	RC-deficient and secretes lauric acid	*ΔglgC::aphII*, *ΔfadD::*P*_cpcBA_-fatB1-aadA*

*Antibiotic-resistance markers confer resistance to spectinomycin (*aadA*) or kanamycin (*aphII*) (Frigaard et al., 2004). The *cpcBA* promoter drives *fatB1* expression. RC, glucose-equivalent reducing carbohydrate*.

*^a^Transesterifiable C12:0*.

*^b^LAS module*.

### Batch cultivation

The saltwater medium A+ used in batch experiments contained, per liter, 18 g NaCl, 5 g MgSO_4_⋅7H_2_O, 1 g NaNO_3_, 0.6 g KCl, 0.05 g KH_2_PO_4_, 0.03 g Na_2_-EDTA, 0.27 g CaCl_2_, 1 g Trizma base (Tris), 1 mL L^−1^ of 3.89 g L^−1^ FeCl_3_⋅6H_2_O stock in 0.1 N HCl, and 1 mL L^−1^ of P1 metals micronutrient solution. The P1 stock solution contained, per liter, 34.26 g H_3_BO_3_, 4.32 g MnCl_2_⋅4H_2_O, 0.315 g ZnCl, 0.03 g MoO_3_ (85%), 12.15 mg CoCl_2_⋅6H_2_O, and 3 mg CuSO_4_⋅5H_2_O. For A+ medium without nitrogen (−N), NaNO_3_ was replaced by an equimolar amount of NaCl.

Liquid cell cultures were grown using a rotary shaker under constant illumination in an atmosphere of 1% CO_2_, 34°C, and 160 μmol photons m^−2^ s^−1^ (μmol m^−2^ s^−1^ hereafter) photosynthetically active radiation (PAR) in 250-mL Erlenmeyer flasks with soft caps to facilitate gas exchange (VWR, Radnor, PA, USA). Batch flask cultures were grown in quadruplicate and standardized to 2.5 mg L^−1^ chlorophyll *a* at the beginning of each experiment. Pre-cultures were similarly normalized and grown to mid-linear phase (15–25 μg mL^−1^ chlorophyll *a*), whereupon cells were concentrated by centrifugation and resuspended in fresh medium for experimental replicates, which were sampled over a time course.

### Continuous culture

Steady-state physiology was assayed in a photobioreactor (PBR) that maintains constant optical density (turbidostasis) over a range of light intensities delivered by 630 and 680 nm light-emitting diodes (LEDs), as described previously (Melnicki et al., 2013). For maximal light penetration and steady-state illumination, cell cultures were maintained at 0.08 OD_730_ in A+ medium containing 0.9 g L^−1^ NH_4_Cl as the nitrogen source, and Tris was omitted as pH 7.5 was maintained independently. Cultures were held at 30°C and constantly sparged with N_2_ gas containing 1.3% CO_2_ at 4.1 L min^−1^. Doubling times were calculated by ln(2)/dilution rate, O_2_ evolution by percent air saturation, photophysiology by pulse amplitude modulation (PAM) fluorometry, and biochemical composition were measured in WT, SA01, and Δ*glgC* strains at light intensities of 5/5 (33), 10/10 (66), 15/15 (99), 20/20 (132), 25/25 (165), 40/40 (264), 60/60 (396), 70/70 (462), 125/60 (610), and 170/60 (759) as incident 630/680 nm light each and (in parentheses) total spherical μmol m^−2^ s^−1^. A linear 2π incident sensor was used to measure individual wavelengths, and total spherical illumination was reported by a 4π sensor. Absorbance scans of total cell culture were measured over a 350–900 nm spectral range using a Shimadzu BioSpec 1601 spectrophotometer (Shimadzu, Kyoto, Japan). Non-transmitted fractions of 630 and 680 nm light were calculated using transmitted light values obtained *in situ* from the linear sensor and normalized to total spherical irradiance as described previously (Melnicki et al., 2013).

### Biochemical analyses

Chlorophyll *a* was measured by absolute methanol extraction of a 1-mL cell pellet and calculated as described previously (Meeks and Castenholz, 1971; Porra et al., 1989). Reducing carbohydrates (RCs) were measured as glucose equivalents by a colorimetric anthrone–sulfuric acid assay described previously (Meuser et al., 2012).

Dry cell weight (DCW) of batch cultures was measured from 2 mL of liquid culture concentrated by centrifugation. The cell pellet was washed once in 1 g L^−1^ Tris buffer (TB), resuspended in 1 mL TB, thoroughly dried at 80°C, and the dry weight of 1 mL TB subtracted to give DCW. From PBR cultures, DCW is represented as ash-free weight from 400 mL steady-state culture concentrated by centrifugation, resuspended in distilled water, dried at 105°C, and burned at 550°C for 1 h. Ash-free weight was calculated as mass lost between drying and burning.

Organic acids (OAs) were quantified by HPLC (Surveyor Plus, Thermo Scientific, Waltham, MA, USA) using 0.45-μm filtered supernatant from −N cultures. A 25-μL sample was injected onto a 150 mm × 7.8 mm fermentation monitoring column (BioRad, Hercules, CA, USA) at 0.5 mL min^−1^ 8 mM H_2_SO_4_ eluent, 45°C column operating temperature, and 50°C refractive index (RI) detector operating temperature, in parallel with a photodiode array detector for absorbance at 210 nm. A standard mix of acetate, pyruvate, succinate, α-ketoglutarate, and α-ketoisocaproate was used for quantification, and all samples were held at 10°C in a thermostated sample tray before injection.

Fatty acyl content was measured as transesterifiable fatty acid methyl esters (FAMEs) using an adapted method (Radakovits et al., 2011). Briefly, 0.5 mL of liquid culture was hydrolyzed and lipids saponified at 100°C for 2 h in 1 mL 95:5% v/v absolute methanol:0.8 g L^−1^ KOH (in H_2_O), after which 1.5 mL 94.2:5.8% v/v methanol:12N HCl was added for acid-catalyzed methylation at 80°C for 5 h. FAMEs were extracted into 1 mL *n*-hexane and the extract was analyzed using an Agilent 7890A gas chromatograph (GC) and DB-5ms column with flame ionization detection (Agilent Technologies, Santa Clara, CA, USA). A flow rate of 1.15 mL min^−1^ H_2_ carrier gas was used to separate FAMEs at 20°C min^−1^ to 230°C, held for 1 min, then 20°C min^−1^ to 310°C and held for 5 min. A standard mix of FAMEs was used for quantification and retention time correlation (37-component FAME mix, Supelco, Bellefonte, PA, USA). Due to insufficient resolution between unsaturated C18 FAs, the combined contents of 18:1, 18:2, and 18:3 are reported as 18:*n*. The unknown (unk) compound that elutes prior to C16:1 was not included in FAME tabulations. A two-tailed *t*-test was performed to determine statistical significance (*p*-value). Lauric acid methyl ester (C12 FAME) was identified via mass spectral analysis conducted using a Varian 3800 GC and Varian 1200 quadrupole MS/MS (Agilent Technologies, Santa Clara, CA, USA) equipped with a Rxi-5ms column (30 mm × 0.25 mm; 0.25 μm film thickness) (Restek Corporation, Bellefonte, PA, USA). A flow rate of 1.2 mL min^−1^ He carrier gas was used to separate FAMEs at 20°C min^−1^ from 70 to 230°C for a 1-min hold, then 20°C min^−1^ to 310°C for a 5-min hold. Mass spectra were obtained after electron ionization at 70 eV. Results were compared to the known mass spectrum of C12 FAME (NIST Mass Spec Data Center, and Stein, 2015).

### Pulse amplitude modulation fluorometry

Variable chlorophyll fluorescence was measured using PAM fluorometry in a DUAL-PAM-100 system (Walz GmbH, Effeltrich, Germany) with a photodiode detector and RG665 filter (Schreiber, 1986). Red measuring light (620 nm) at the lowest power was pulsed at 1000 Hz during the dark and at 10,000 Hz during 635 nm actinic illumination at 98 μmol m^−2^ s^−1^. From PBR cultures, 3 mL was immediately transferred to a cuvette and fluorescence induction was measured with a programed script consisting of 15 s darkness, 30 s actinic illumination (O), application of a saturating pulse at 2000 μmol m^−2^ s^−1^ for 200 ms (J), 5 s of only far-red light (730 nm) (I), another 15 s of actinic light (P), and 30 s of darkness (S). Variable fluorescence observed during the O-J-I-P-S induction provided the basis to compare changes in the electron transport processes downstream of PSII. The effective quantum yield of PSII (YII’) was measured by transient fluorescence changes between “J” and “I” states. The estimated redox status of the plastoquinone (PQ) pool was determined by the rise from “I” to “P” level, normalized to the total variable fluorescence observed over this period, and subtracted from 1 (Chylla and Whitmarsh, 1989). Relative changes in electron transport downstream of the PQ pool were measured by P > > S quenching as the drop from “P” to “S” states relative to the variable fluorescence (Serrano et al., 1981). The relative dark rate of PQ oxidation was obtained from the declining slope of post-illumination fluorescence, calculated from between 10 and 20 s after the level had peaked (Ryu et al., 2003). Dark-adapted measurements were taken after cells were held in the dark for 20 min and then acclimatized in actinic light for 90 s before induction.

## Results

### Batch culture productivity

**Figure 1:**
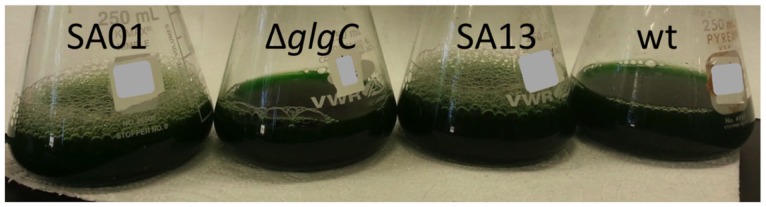
**Foaming is visible atop culture medium of the lauric acid-secreting *Synechococcus* sp. PCC 7002 strains SA01 and SA13**.

Nitrogen replete and nitrogen deplete batch cultures of WT, SA01, Δ*glgC*, and SA13 were analyzed over 48 h for chlorophyll *a*, DCW, FAME, RC, and OA. Cultures of C12-secreting strains developed a layer of surfactant bubbles (Figure 1).

#### Chlorophyll *a* and dry cell weight

Bulk biomass accumulation in nutrient replete batch cultures yielded an increase in chlorophyll *a* content of 12- to 15-fold over 48 h (Figure S1A in Supplementary Material), and DCW accumulated 4- to 5-fold (Figure S1C in Supplementary Material). In nitrogen-deplete cultures, growth attenuation was suggested by unchanging chlorophyll *a* content and DCWs that were within the range of error relative to inoculum DCWs over the time course (Figures S1B,D in Supplementary Material).

#### Fatty acids

**Figure 2:**
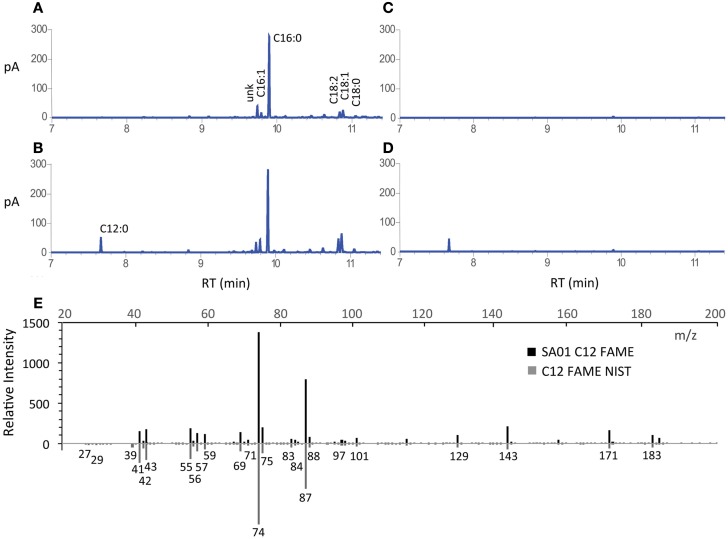
**FAME profiles and identification of secreted FA from batch cultivation of WT and SA01 strains of *Synechococcus* sp. 7002**. GC-FID chromatograms of FAME from representative **(A)** WT and **(B)** SA01 cell culture, and **(C)** WT and **(D)** SA01 cell-free supernatant. **(E)** Mass spectrum from GC–MS of SA01 FAME matching lauric acid methyl ester from the NIST database (C12 FAME NIST) (NIST Mass Spec Data Center, and Stein, 2015). The labeled standard is shown on the mirror axis. m/z, mass-to-charge ratio; pA, picoamps (detection current); RT, retention time.

**Figure 3:**
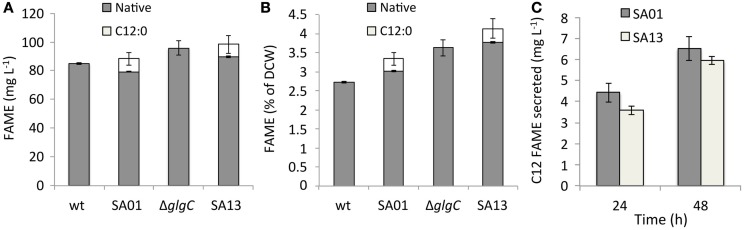
**FAME content of nutrient replete batch cultures after 48 h represented (A) by volume and (B) as a function of average dry cell weight (DCW)**. Native, endogenous fatty acyls; C12:0, heterologous lauric acid. **(C)** C12 FAME recovered from the medium of nutrient replete SA01 and SA13 cultures after 24 and 48 h. Error bars represent SD over four biological replicates.

Secretion conferred by the LAS modification of transesterifiable C12 FAs into batch culture medium is demonstrated in Figures 2A–D. The identity of transesterified C12 was confirmed by GC–MS (Figure 2E). Lauric acid was detected neither during nitrogen starvation nor when *fatB1* was expressed at a neutral locus without concurrent deletion of the putative AAS *fadD* (not shown). After 48 h, total FAME recovered from nutrient replete batch cultures reached 85.0 ± 0.7 (WT), 83.6 ± 4.5 (SA01), 95.3 ± 5.0 (Δ*glgC*), and 93.6 ± 6.1 (SA13) mg L^−1^ (Figure 3A), representing 3–5% of DCW (Figure 3B). Over 48 h, SA01 and SA13 generated, respectively, 9.1 ± 0.4 and 8.7 ± 0.6 mg L^−1^ C12, accounting for ~10% of total FAME (Table 2). C12 was recovered from culture medium of SA01 and SA13, respectively, at concentrations of 4.4 ± 0.4 and 3.5 ± 0.2 mg L^−1^ after the first 24 h, and 6.5 ± 0.6 and 5.9 ± 0.2 mg L^−1^ after 48 h (Figure 3C), an ~70% secretion level in both strains.

**Table 2:** **Percent of total FAME by chain length from 48-h nutrient replete cultures, corresponding to Figure 3**.

	% of total FAME				
Strain	12:0	16:0	16:1	18:0	18:*n*
wt	nd	53.1 ± 0.8	11.8 ± 0.3	1.7 ± 0.1	33.4 ± 0.6
SA01	10.9 ± 0.4^a^	50.2 ± 0.9	7.3 ± 0.3	3.1 ± 0.4	28.4 ± 0.6
	7.8 ± 0.4^b^				
*ΔglgC*	nd	53.5 ± 1.1	10.2 ± 0.6	3.6 ± 0.7	32.6 ± 0.7
SA13	9.0 ± 0.5^a^	48.6 ± 0.2	8.3 ± 0.5	3.2 ± 1.1	30.8 ± 0.5
	6.3 ± 0.2^b^				

*The two values for C12:0 represent total (a) and secreted (b) FAs and are not additive. FAMEs are derived from lauric (12:0), palmitic (16:0), palmitoleic (16:1), stearic (18:0), oleic (18:1), linoleic (18:2), and α-linolenic (C18:3) FAs. Unsaturated C18 FA content is grouped (18:*n*). Error is represented as SD over four biological replicates*.

*^a^Cell culture*.

*^b^Cell-free supernatant of ^a^*.

*nd, none detected*.

Distribution of FAME in 48-h nutrient replete cultures (Table 2) is consistent with previous studies of *Synechococcus* sp. 7002, including cumulative levels of unsaturated C18 FA (Kenyon, 1972; Sakamoto et al., 1997; Sakamoto and Bryant, 2002). Strains with the LAS modification showed diminished contents of 16:0 (*p* < 0.02), 16:1 (*p* < 0.001), and 18:*n* (*p* < 0.01). All three mutants contained twofold more 18:0 than WT. The Δ*glgC* mutant exhibited less 16:1 than WT (*p* < 0.02), while 16:0 and 18:*n* occurred at WT levels. In the AGPase-disrupted background, the LAS modification conferred a lower fraction (*p* < 0.02) of C12 relative to total FAME under the culturing conditions used.

#### Carbohydrates and organic acids

**Figure 4:**
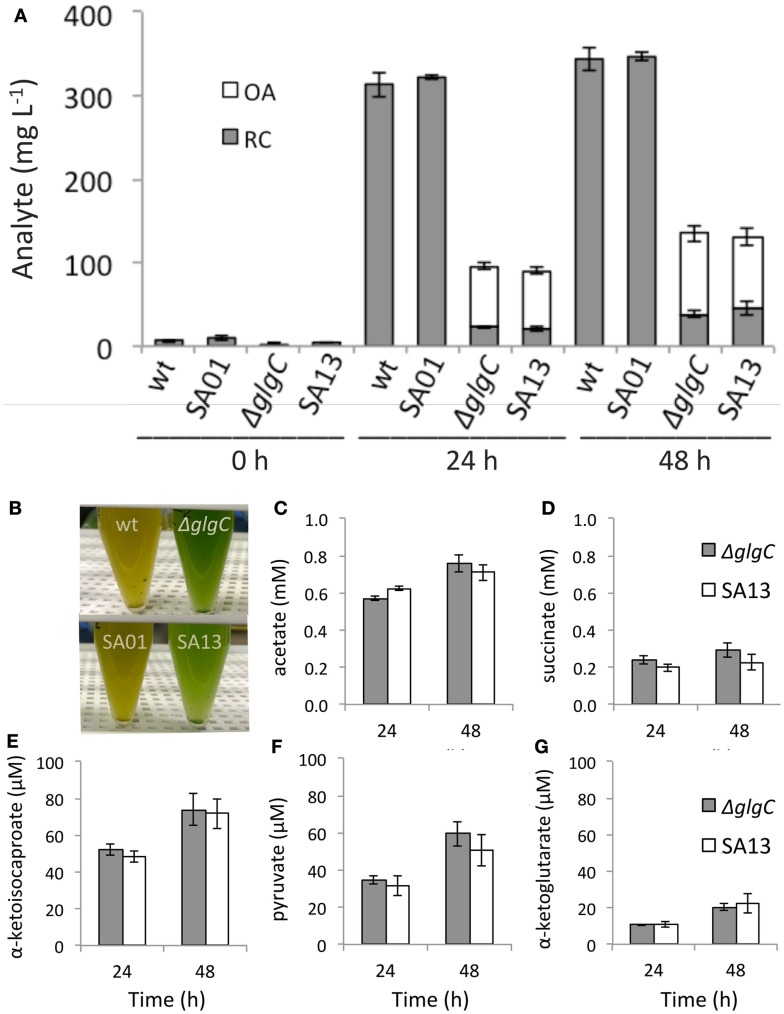
**(A)** Reducing carbohydrate (RC) content and secreted organic acids (OAs) by volume in batch cultures of WT, SA01, Δ*glgC*, and SA13 during nitrogen deprivation at inoculation (0 h) and after 24 and 48 h. **(B)** Pigmentation differences between wild-type and carbohydrate-deficient backgrounds during nitrogen deprivation. **(C–G)** Individual OA secreted from Δ*glgC* and SA13 strains after 48 h of nitrogen deprivation. Error bars represent SD over four biological replicates.

During nitrogen deprivation (−N), AGPase-disrupted strains accumulated substantially less RC than the WT background: over 24–48 h, RC on a culture volume basis reached 7–11% of WT levels in Δ*glgC* and 6–13% in SA13 (Figure 4A). Under the same conditions, RC comprised 30% of DCW in the WT background after 24 h and remained at this percentage over the next 24 h; whereas RC was accumulated to 4–5% of DCW in Δ*glgC* and 4–7% in SA13. The carbohydrate-deficient background secreted OA during −N to a total of 15% of DCW in both Δ*glgC* and SA13 after 24 and 48 h. Neither WT nor SA01 secreted detectable amounts of OA over the −N time course, and OAs were not detected in nutrient replete growth media (not shown). Combined, RC and OA (RC + OA) in the AGPase-disrupted strains accumulated under −N conditions to 19% of DCW. Relative to WT RC levels, RC + OA in Δ*glgC* cultures reached 31% after 24 h and 39% after 48 h, and SA13 reached 28 and 38% of WT, respectively (Figure 4A). While WT and SA01 cultures developed yellow coloration during −N, the AGPase-disrupted cultures did not (Figure 4B). Under the culturing conditions used, acetate was the most abundant OA secreted from the carbohydrate-deficient strains during −N, followed by succinate and α-ketoisocaproate (Figures 4C–E). Lesser concentrations of pyruvate and α-ketoglutarate were also observed (Figures 4F,G). Levels of OA secretion were not affected by the LAS modification.

### Steady-state physiology

At stable growth rate for each indicated light intensity in the LED-PBR, cultures of *Synechococcus* sp. 7002 WT, SA01, and Δ*glgC* were analyzed for RC, DCW, FAME, O_2_ evolution, doubling rate, and photophysiological characteristics. Growth rate and O_2_ production measurements for the same conditions were made previously using a separate cultivar of WT *Synechococcus* sp. 7002 (not shown), which demonstrate the reproducibility of PBR measurements (Work, 2014).

#### Biomass profiles

**Figure 5:**
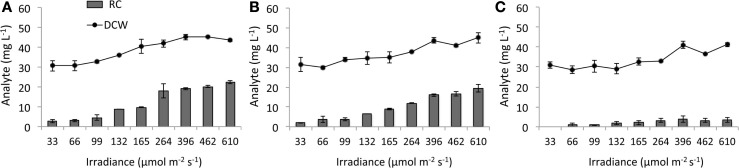
**Dry cell weight (DCW) and reducing carbohydrates (RCs) in steady-state PBR cultures**. **(A)** WT, **(B)** SA01, and **(C)** Δ*glgC* strains of *Synechococcus* sp. 7002 with increasing 630/680 nm light intensity reported as spherical irradiance. The 33 μmol m^−2^ s^−1^ RC value for Δ*glgC* and 759 μmol m^−2^ s^−1^ biomass values were not available. Error bars represent SD over three samplings.

Disruption of AGPase inhibited RC accumulation while RC levels in the WT background increased with light intensity (Figure 5). At 610 μmol m^−2^ s^−1^, RC represented 51% of DCW in WT and 43% in SA01, but Δ*glgC* reached only 10% of DCW which occurred at 264 μmol m^−2^ s^−1^. Due to the dilute concentration of PBR cultures, FAMEs of C16:0 and C12:0 (C12 hereafter) were detectable but not quantifiable (<1 mg L^−1^). In representative PBR cultures at 396 μmol m^−2^ s^−1^, all observed C12 was recoverable from SA01 cell-free filtrate, and C12 was not detected in WT or Δ*glgC* (not shown) (Work, 2014).

#### Growth rates and O_2_ evolution

**Figure 6:**
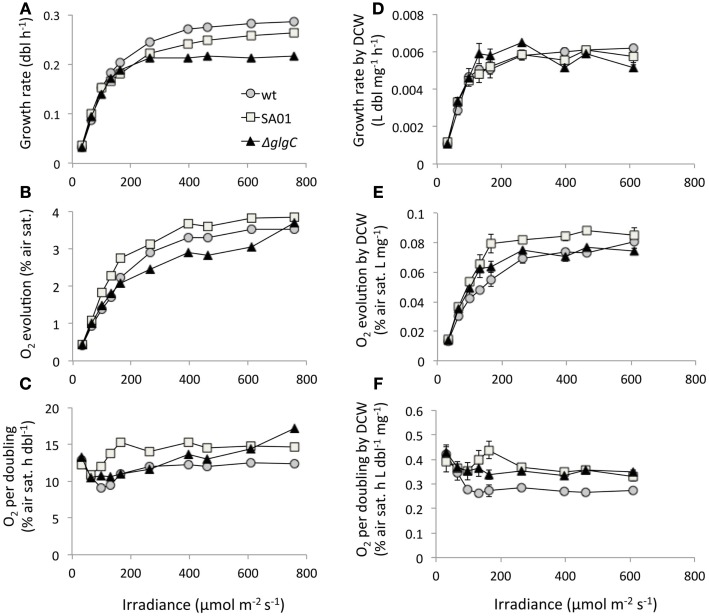
**Growth rates, O_2_ evolution, and dry cell weight (DCW) normalization of steady-state PBR cultures by light intensity**. **(A)** Doubling rate, **(B)** bulk O_2_ evolution, **(C)** O_2_ production normalized to growth rate, **(D)** doubling rate per unit DCW, **(E)** O_2_ evolved on the basis of DCW, and **(F)** per-doubling O_2_ evolution normalized to DCW. Values for DCW at 759 μmol m^−2^ s^−1^ were not available. Light intensity is reported as spherical irradiance. Error bars represent SD over three samplings at each light intensity.

Minimum stable doubling times of 3.5 h (WT), 3.8 h (SA01), and 4.6 h (Δ*glgC*) were observed at 759 μmol m^−2^ s^−1^ in the WT background and at 462 μmol m^−2^ s^−1^ in Δ*glgC* (Figure 6A). Bulk O_2_ evolved by SA01 exceeded both WT and Δ*glgC* over the majority of light intensities tested (Figure 6B). On a per-doubling basis, Δ*glgC* produced O_2_ at levels similar to WT and in fact surpassed WT at 396 μmol m^−2^ s^−1^ and above (Figure 6C) despite diminished growth rates (Figure 6A). The doubling rate required per unit DCW was similar between all strains (Figure 6D). O_2_ evolved by Δ*glgC* was comparable to WT by bulk DCW (Figure 6E) but greater on the basis of DCW-normalized growth rate (Figure 6F). The uncoupling of O_2_ evolution from growth rate in Δ*glgC* at high irradiance was not observed when further normalized to DCW (Figure 6F). Despite attaining lower growth rates than WT at 264 μmol m^−2^ s^−1^ and above, SA01 exhibited consistently elevated O_2_ evolution by volume (Figure 6B), growth rate (Figure 6C), bulk DCW (Figure 6E), and DCW-normalized growth rate (Figure 6F).

#### Photophysiology

**Figure 7:**
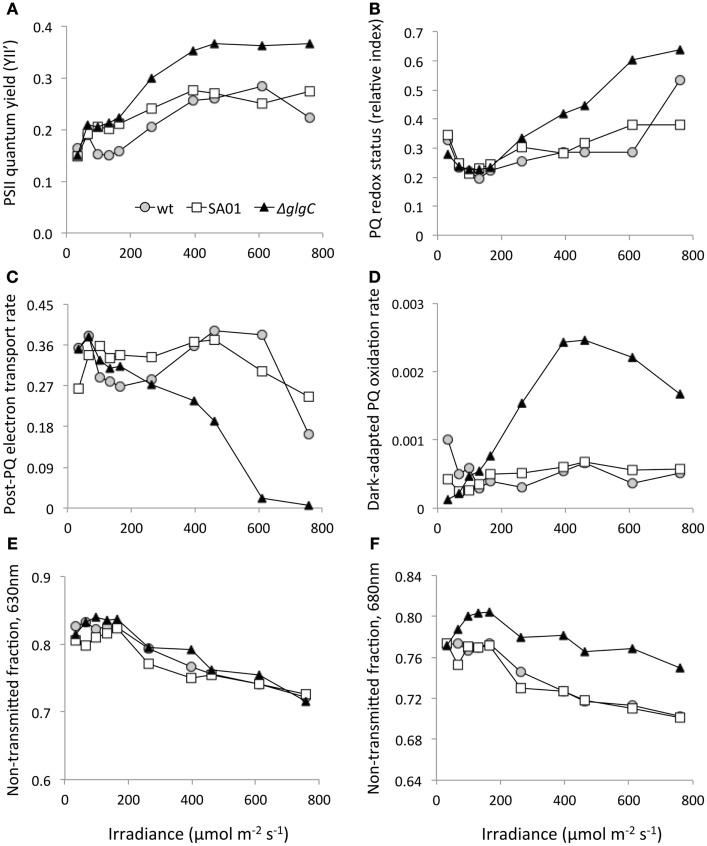
**Pulse amplitude modulation (PAM) fluorometry from steady-state PBR cultures of WT, SA01, and Δ*glgC* over a range of light intensities**. **(A)** PSII quantum yield (YII’). **(B)** Relative redox status of the PQ pool (more positive is more reduced). **(C)** Relative P > > S electron transport rates downstream of PQ. **(D)** Dark PQ oxidation rates in dark-adapted cultures. Non-transmitted fractions of **(E)** 630 nm and **(F)** 680 nm light by cell culture.

Photosynthetic electron transport appears to be altered by AGPase disruption (Figure 7). With increasing irradiance of the Δ*glgC* culture, a higher quantum yield of PSII was observed (Figure 7A), and the PQ pool became more reduced (Figure 7B) than the WT background. The rate of electron transport downstream of the PQ pool was also adversely affected by *glgC* disruption (Figure 7C), as less P > > S quenching occurred in this background with higher light. After dark adaptation, Δ*glgC* cultures exposed to 165 μmol m^−2^ s^−1^ and above exhibited more rapid rates of PQ oxidation in the dark (Figure 7D). The transmittance of 630 nm light by cell cultures was unaffected between strains (Figure 7E), but Δ*glgC* transmitted less 680 nm light than the WT background (Figure 7F) indicating more absorption or scattering by the strain at this wavelength.

## Discussion

Derived from photosynthetically fixed CO_2_, FAs secreted by genetically engineered cyanobacteria have yielded up to 197 mg L^−1^ FFA by *Synechocystis* sp. 6803 and 131 mg L^−1^ FFA (6.5 mg L^−1^ d^−1^) by *Synechococcus* sp. 7002 (Liu et al., 2011; Ruffing and Jones, 2012; Ruffing, 2014). Secretion of 4.4 mg L^−1^ day^−1^ transesterifiable lauric acid (C12) from modified strains of *Synechococcus* sp. 7002 was achieved in batch cultures that grew at a similar rate to WT. Sodium lauryl sulfate is a common ingredient in soap, and the foam layer atop cultures secreting C12 suggests detergent activity. Under these conditions, C12 may accumulate in surface bubbles. Phase separation may be a consideration in applying photosynthetic FA secretion on an industrial scale, and actively removing C12 from cultures, for example by hexane overlay (Davies et al., 2014) or solid-state methods (Léonard et al., 2011), may create more favorable conditions for productivity.

Attenuating the synthesis of polymeric carbohydrates did not augment C12 production during normal growth, and attempts to direct metabolism to FAs by nitrogen starvation instead eliminated C12 altogether. The absence of C12 may be due to cessation of protein and/or lipid synthesis under these conditions, or, since a phycocyanin-related promoter is responsible for *fatB1* expression, the gene may be downregulated in times of nitrogen stress, as phycobiliproteins can be degraded as an intracellular nutrient source (Sauer et al., 1999; Richaud et al., 2001). Additionally, the AGPase-disrupted batch cultures exhibited a non-bleaching phenotype when nitrogen-deprived, and as previously reported, higher absorbances in the 580–650 nm phycobilin range suggest that these proteins are not deconstructed for nutrients in this background as they are in WT (Guerra et al., 2013; Davies et al., 2014). Similar characteristics were described in carbohydrate-deficient mutants of *Synechocystis* sp. 6803 and *Synechococcus elongatus* 7942 (Carrieri et al., 2012; Gründel et al., 2012; Hickman et al., 2013).

Intracellular carbohydrate accumulation during nitrogen stress requires AGPase for glucose polymerization in *Synechococcus* sp. 7002 (Davies et al., 2014), *S. elongatus* PCC 7942 (Hickman et al., 2013), and *Synechocystis* sp. 6803 (Carrieri et al., 2012; Gründel et al., 2012), and energy spilling in the form of OA secretion was observed upon disruption of this function; and a similar outcome occurred with glycogen synthase deletions (Xu et al., 2013). Of the OA secreted by nitrogen-deprived Δ*glgC* and SA13 strains, pyruvate (C_3_), α-ketoglutarate (C_5_), and succinate (C_4_) are also gluconeogenic metabolites (Zhang and Bryant, 2011; Steinhauser et al., 2012). In *S. elongatus* 7942, α-ketoglutarate has been demonstrated as an effector of the nitrogen regulator NtcA (Vázquez-Bermúdez et al., 2001; Tanigawa et al., 2002). Possibly derived from protein degradation or metabolite redistribution, α-ketoisocaproate (C_6_) is a biosynthetic intermediate of the amino acid leucine and can be converted to acetyl-CoA and acetoacetate, which, along with pyruvate and acetate (C_2_), are direct precursors of FAs, terpenoids, higher alcohols, and reduced storage polymers such as poly-3-hydroxybutyrate (PHB) and polyhydroxyalkanoate (PHA). Though biosynthetic enzymes for the latter two have not been identified in *Synechococcus* sp. 7002 (McNeely et al., 2010), secreted OA could be supplied to a capable organism by medium exchange (Niederholtmeyer et al., 2010) or co-cultivation (Contag, 2012; Therien et al., 2014).

Steady-state photoautotrophic doubling times of 3.5 h (WT), 3.8 h (SA01), and 4.6 h (Δ*glgC*) are close to the fastest observed in *Synechococcus* sp. 7002 (Ludwig and Bryant, 2012). Increased O_2_ production on the basis of DCW-normalized growth rate in both SA01 and Δ*glgC* may represent unidentified photosynthetic energy sinks (Badger et al., 2000; Nomura et al., 2006; Suzuki et al., 2010; Zhu et al., 2010; Xu et al., 2013), which in SA01 could be related to the synthesis of secreted FA. Diminished photosynthetic productivity caused by AGPase disruption was evident, as Δ*glgC* reached maximum growth rate at a lower light intensity than WT. After growth rate saturation, dissipation of excess radiant energy appears to be accomplished in part by RC storage in the WT background. Restricting RC by AGPase disruption resulted in a more reduced, less oxidizable PQ pool indicating overreduction of the photosynthetic electron transport chain and/or the inability to utilize photosynthetic reductant. However, high rates of PQ oxidation by Δ*glgC* in the dark possibly demonstrate a respiratory or other continuous quenching function (Joët et al., 2002; Bailey et al., 2008; McDonald et al., 2011). The severity of these redox alterations may lead to the protection of PSII in Δ*glgC* under irradiances at which WT and SA01 accumulated RC, as evidenced by O_2_ evolution decoupling, elevated PSII quantum yields, and more scattered or absorbed 680 nm light, perhaps owing in part to increased content of PSII or a phycobiliprotein such as phycocyanobilin that can absorb at 680 nm and is involved in free radical scavenging (Alvey et al., 2011; Ge et al., 2013). Demonstrating a robust capacity to manage excess light energy, *Synechococcus* sp. 7002 could be a promising organism in scaled systems (Dong et al., 2009; Zhu et al., 2010; Ludwig and Bryant, 2012), and efforts to reroute metabolic flux may identify enzyme targets through further investigation of carbon partitioning at high light in the present genetic backgrounds.

The planktonic cyanobacterium *Synechococcus* sp. 7002 was engineered to convert photosynthate into biofuel precursors, which were naturally secreted from the cell. Lauric acid and OAs can be processed into diesel and alcohols or used as a carbon source for other organisms, and their recovery from culture filtrate avoids costly cell harvesting and lysis. Though redirection of carbohydrate-deficient metabolism toward FA synthesis was not effective under the present conditions, central metabolites for FA, terpenoid, and glucan biosynthesis were generated that potentially could be captured with further metabolic adjustments for redistribution into desired pathways.

## Author Contributions

The LAS module was constructed by VW, who also transformed and developed the LAS strains, performed batch cultivations and biochemical assays, and compiled this manuscript. MM performed PAM fluorometry measurements and contributed to PBR research design. EH designed, built, and operated the LED-PBR to generate O_2_ and dilution rate measurements, and assisted with dry weight measurements. FD performed mass spectral analysis, identified and helped quantify OAs, and assisted with quantitation of RCs. LK contributed to PAM fluorometry data collection. AB and MP were responsible for the project’s conception and provided laboratory resources. All authors revised the manuscript for intellectual content.

## Conflict of Interest Statement

The authors declare that the research was conducted in the absence of any commercial or financial relationships that could be construed as a potential conflict of interest.

## Supplementary Material

The Supplementary Material for this article can be found online at http://journal.frontiersin.org/article/10.3389/fbioe.2015.00048/abstract

## Abbreviations

ADP, adenosine diphosphate; DCMU, 3-(3,4-dichlorophenyl)-1,1-dimethylurea; LED, light-emitting diode; PCC, Pasteur culture collection.
