# Supplementary figures and images for: Lauric Acid Production in a Glycogen-Less Strain of Synechococcus sp. PCC 7002
Source: Front Bioeng Biotechnol. 2015 Apr 24;3:48. doi: 10.3389/fbioe.2015.00048 (PMC4408914; doi:10.3389/fbioe.2015.00048)

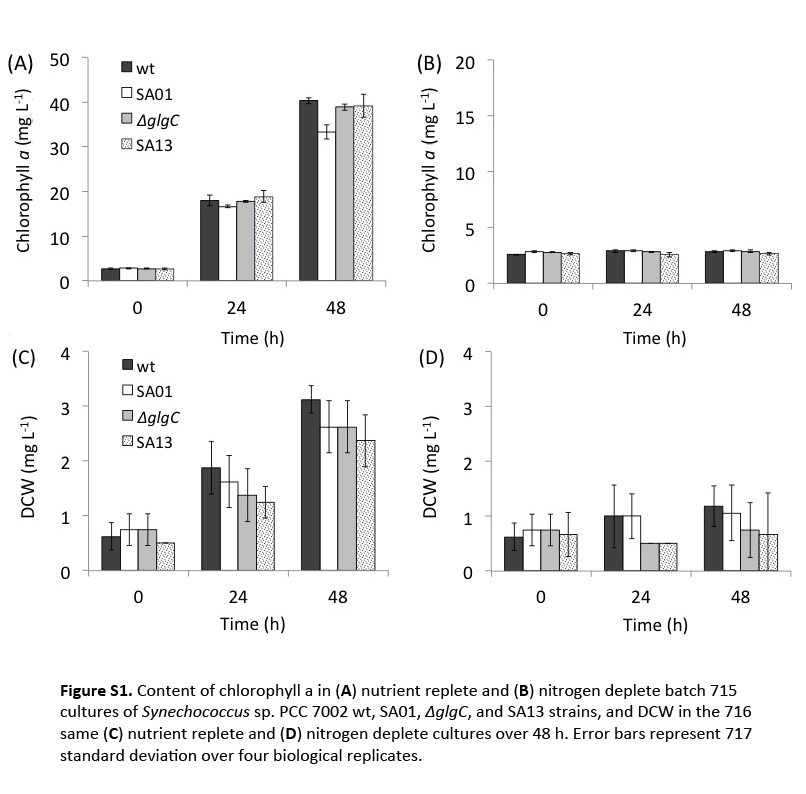

Supplement: Supplementary file 1 [file Image_1.JPEG]
